# Temporal and region‐specific variations in genome‐wide inbreeding effects on female size and reproduction traits of rainbow trout

**DOI:** 10.1111/eva.13308

**Published:** 2021-10-21

**Authors:** Katy Paul, Jonathan D'Ambrosio, Florence Phocas

**Affiliations:** ^1^ Université Paris‐Saclay INRAE AgroParisTech GABI Jouy‐en‐Josas France; ^2^ SYSAAF Station INRAE‐LPGP Campus de Beaulieu Rennes France

**Keywords:** dominance, fish, inbreeding depression, overdominance, run of homozygosity, selection

## Abstract

Recent studies have shown that current levels of inbreeding, estimated by runs of homozygosity (ROH), are moderate to high in farmed rainbow trout lines. Based on ROH metrics, the aims of our study were to (i) quantify inbreeding effects on female size (postspawning body weight, fork length) and reproduction traits (spawning date, coelomic fluid weight, spawn weight, egg number, average egg weight) in rainbow trout, and (ii) identify both the genomic regions and inbreeding events affecting performance. We analysed the performance of 1346 females under linear animal models including random additive and dominance genetics effects, with fixed covariates accounting for inbreeding effects at different temporal and genomic scales. A significant effect of genome‐wide inbreeding (*F*) was only observed for spawning date and egg weight, with performance variations of +12.3% and −3.8%, respectively, for 0.1 unit increase in *F* level. At different local genomic scales, we observed highly variable inbreeding effects on the seven traits under study, ranging from increasing to decreasing trait values. As widely reported in the literature, the main scenario observed during this study was a negative impact of recent inbreeding. However, other scenarios such as positive effects of recent inbreeding or negative impacts of old inbreeding were also observed. Although partial dominance appeared to be the main hypothesis explaining inbreeding depression for all the traits studied, the overdominance hypothesis might also play a significant role in inbreeding depression affecting fecundity (egg number and mass) traits in rainbow trout. These findings suggest that region‐specific inbreeding can strongly impact performance without necessarily observing genome‐wide inbreeding effects. They shed light on the genetic architecture of inbreeding depression and its evolution along the genome over time. The use of region‐specific metrics may enable breeders to more accurately manage the trade‐off between genetic merit and the undesirable side effects associated with inbreeding.

## 1 INTRODUCTION

Inbred offspring tend to have a greater number of abnormalities and poorer survival, growth and fertility compared to the progeny of unrelated parents (Fessehaye et al., 2007; Mrakovčič, & Haley, 1979; Thrower & Hard, 2009). This decrease in fitness is referred to as inbreeding depression and is caused by the increased homozygosity of individuals (Aulstad & Kittelsen, 1971; Charlesworth & Charlesworth, 1999; Landweber & Dobson, 1999). Inbreeding depression is currently explained by two main hypotheses. The partial dominance hypothesis assumes that inbreeding depression results from the expression of deleterious recessive alleles in homozygous individuals, that is the so‐called genetic load. These deleterious alleles are present at low frequencies in populations. As inbreeding increases in a population, the frequency of deleterious recessive homozygotes, which were initially hidden in heterozygotes, increases and exposes the deleterious effects (Fu & Ritland, 1994). Alternatively, the overdominance hypothesis refers to dominance effects displaying the heterozygote advantage and, as inbreeding increases, the number of heterozygous genotypes is reduced and the superior heterozygote genotypes are less frequent (Wright, 1984). A final hypothesis is that epistasis between dominance effects across loci can also generate inbreeding depression, such that as inbreeding accrues, favourable gene combinations among heterozygous genotypes decrease in frequency (Jain & Allard, 1966). Distinguishing this ‘pseudo‐overdominance’ hypothesis from true overdominance is hampered by the difficulty in distinguishing between linked deleterious mutations in a genome region and a single locus with heterozygote advantage (Ohta & Kimura, 1969). If deleterious mutations are common, genome regions may often carry mutations in different genes in repulsion. The region would therefore display a heterozygote advantage even though no overdominant gene is present. The partial dominance hypothesis is widely accepted as the most common mechanism causing inbreeding depression and has received the strongest empirical support until now (Ceballos et al., 2018; Charlesworth & Willis, 2009; Fu & Ritland, 1994). All hypotheses concern the existence of genetic effects that are not additive and need to be considered when describing performance. Fitness‐related traits are more affected by inbreeding than traits under less severe directional selection (DeRose & Roff, 1999). Inbreeding depression has been documented in many different plants and animals, both in the wild (Chapman et al., 2009; Reed & Frankham, 2003) and among farmed livestock (Leroy, 2014). In populations under artificial selection, an accumulation of inbreeding is unavoidable because of the use of a limited number of breeding individuals and their intense directional selection. These procedures lead to the widespread use of related individuals as parents of the next generation, thus reducing the effective population size and hence genetic diversity over the generations. In livestock species, inbreeding depression has a direct impact on the income of breeders (Leroy, 2014) and maintaining genetic diversity is a challenge.

Before the massive increase in genomic information, pedigree relationships were used to estimate inbreeding effects and manage inbreeding within a population. The effect of inbreeding on economically important traits was mainly investigated by regressing phenotypes of interest on the inbreeding coefficient value using pedigree (Wang et al., 2002), which severely limited the understanding of inbreeding effects. Pedigree information is based on the expected proportion of the genome that is identical by descent (IBD) between two parents, so it does not capture variations due to Mendelian sampling and linkage during gamete formation (Kardos et al., 2015; Wang, 2016). Genomic information enables an estimate of the realized proportion of the genome shared by two individuals either genome‐wide or in specific regions (Hill & Weir, 2011). The use of genomics rather than pedigrees to measure inbreeding has therefore been promoted (Kardos et al., 2015; Wang, 2016) as enabling major advances in our understanding of inbreeding and identifying the regions and genes that result in inbreeding depression along the genome (Kardos et al., 2016; Pryce et al., 2014). It has also led to renewed interest in estimates of dominance effects that can improve our understanding of the genetic architecture of inbreeding depression and enable more accurate estimates of inbreeding effects (Toro & Varona, 2010; Vitezica et al., 2016).

Although access to local inbreeding is now greatly facilitated due to the development of genomics, the number of studies focusing only on global inbreeding and local inbreeding remains quite low (Botero‐Delgadillo et al., 2020; Judson et al., 2018; Lieutenant‐Gosselin & Bernatchez, 2006). The use of region‐specific metrics to identify areas of low genetic diversity may enable breeders to more accurately manage the trade‐off between genetic merit and the undesirable side effects associated with inbreeding (Howard et al., 2017).

Among the various molecular‐based inbreeding indicators available (Ferencakovic et al., 2013; Zhang et al., 2015), runs of homozygosity (ROH) have been revealed as being the most accurate measure of inbreeding (Howrigan et al., 2011; Zhang et al., 2015). ROH are defined as contiguous homozygous stretches of the genome assumed to be inherited from a common ancestor and thus considered as IBD segments (McQuillan et al., 2008). ROH detection has been used to identify genetic anomalies based on homozygosity mapping (Keller & Wallers, 2002; Li et al., 2011). In both humans and cattle, it has been found that ROH are enriched with deleterious variants (Szpiech et al., 2013; Zhang et al., 2015) and they can be used to directly estimate inbreeding depression (Silió et al., 2013). Keller et al. (2011) showed by simulation that ROH‐based inbreeding measure is the most powerful method to detect inbreeding depression (compared to pedigree or marker‐by‐marker‐based inbreeding metrics) and that the statistical power of ROH‐based inbreeding metrics was good (80%) in a randomly breeding population of moderate effective population size (i.e. 100) with only a small sample size of 700 individuals.

As well as its ability to measure region‐specific inbreeding, ROH is able to date inbreeding events through the length of ROH segments (Ceballos et al., 2018; Gomez‐Raya et al., 2015). A large ROH is likely to result from a recent inbreeding event because only a few recombinations have occurred since generation of the most recent common ancestor of the parents, while a small ROH is in favour of an older inbreeding event as several recombination events are likely to have occurred, leading to reduced IBD segments (Ceballos et al., 2018; Purfield et al., 2012). In addition, when Ne is small, rare mutant alleles are tagged by long haplotypes as a result of relatively recent inbreeding. However, when Ne is large, these long haplotypes are broken down by recombination and new ones are not created rapidly because new inbreeding occurs slowly (Keller et al., 2011; Thompson, 2013).

In the particular case of *Oncorhynchus mykiss*, D'Ambrosio et al. (2019) showed that effective population sizes of French farmed rainbow trout lines have been steadily decreasing during the past 10 generations and inbreeding appears to be high not only due to selection, but also because of founder effects and sweepstakes reproductive success at the start of breeding programmes. The impact of these significant levels of inbreeding on French rainbow trout performance needs to be quantified in order to assess potential inbreeding depression phenomena and risk of limiting future genetic gains due to a loss of genetic diversity (Jannink, 2010; Muller & Pearson, 1979). Estimates of inbreeding effects on important traits in fish have been limited to a few experiments on salmonids, as reviewed by Wang et al. (1999, 2002). These previous studies revealed significant but generally moderate inbreeding depression effects on weight at harvest (Kincaid, 1976; Pante et al., 2001; Rye & Mao, 1998), body weight and length (Naish et al., 2013), egg mass, egg hatchability and fry survival (as reviewed by Kincaid, 1983), on egg numbers and the spawning age of rainbow trout (Su et al., 1996) as well as on spawning date for wild populations (Waters et al., 2020).

Therefore, based on ROH metrics, the objectives of our study were to quantify the effects of dominance and inbreeding on female size and reproduction traits in rainbow trout and identify both the genomic regions involved and the time of inbreeding events with effects on these performance traits. The traits under study were therefore female fork length, postspawning weight, spawning date, coelomic fluid weight, spawn weight, egg numbers and egg size. The first question we addressed was whether genome‐wide inbreeding and dominance effects could explain significant proportions of phenotypic variance in female size and reproduction performance. The second issue was to assess whether the effects of inbreeding were mainly due to recent inbreeding events and only observed in specific regions of the genome. The last question was whether our results could corroborate the partial dominance hypothesis, that is the major role of deleterious recessive alleles as the underlying mechanism explaining inbreeding depression.

As far as we know, this is the first study to have reported on genome‐wide and region‐specific inbreeding effects based on ROH metrics, as well as their temporal variations for fish traits. Our results may help rainbow trout breeders to design and implement new genomic selection methods that take account of local genetic diversity along the genome to manage inbreeding at the genome level. In addition, and more globally, they will perhaps shed new light on the genetic architecture of inbreeding depression and its evolution over time.

## 2 MATERIALS AND METHODS

### 2.1 Population

The phenotyped and genotyped rainbow trout population comprised 1346 females from two successive cohorts: C1 (726 individuals) and C2 (620 individuals), which were produced in 2014 and 2015, respectively. After hatching, the juveniles were raised to about 20 g (C1) in size or 58 g (C2) at the Escort site on the plain (spring water temperature within the range 14–16°C) in 17 m^3^ breeding tanks at densities increasing from 2 to 25 kg/m^3^ (C1) or 36 kg/m^3^ (C2). They were then moved from the plain to the mountains at the Sarrance site (spring water temperature within the range 8–9°C) and raised in 126 m^3^ tanks until they reached weights of 450 g (C1) or 580 g (C2). Finally, the trout were transferred to 70 m^3^ tanks with rearing densities ranging from 40 to 100 kg/m^3^ until spawning. Both juveniles and trout were fed with commercial standard diets, although some specific modifications were requested by the breeder regarding the diet given to trout from 1 kg until spawning.

The two cohorts were part of the 9th generation of selection by the ‘Viviers de Sarrance’ breeding company and were produced from 71 fathers and 83 mothers. This 9th generation was the first to have been produced using an optimum contribution method to select and mate the parents for a targeted genetic gain while minimizing the increase in inbreeding through a ‘Minimum Parentage Selection’ procedure (Chapuis et al., 2016). There were around 4 (±2) full‐sibs and 45 (±20) half‐sibs per parent in the phenotyped population under study. Details of the population structure can be found in the article by D'Ambrosio et al. (2020).

### 2.2 Phenotypes

Raw phenotypes were collected from 2‐year‐old females. As for size traits, the ready‐to‐spawn weight (FW in g), postspawning weight (PW in g) and fork length (FL in mm) of the females were recorded (Table 1).

**TABLE 1:** Summary statistics of female reproduction and weight traits at 2 years of age

Traits	Number of individuals	Mean	Standard deviation	Median	Min	Max
Postspawning body weight (g)	1346	1867.0	410.4	1840.0	840.0	3116.0
Female fork length (mm)	1346	525.1	34.9	527.0	430.0	660.0
Spawning date (week rank)	1346	2.6	1.5	2.0	1.0	5.0
Coelomic fluid (g)	1187	43.2	57.3	32.0	1.0	455.0
Spawn weight (g)	1346	189.6	70.9	182.0	20.0	398.0
Egg number (#)	1346	4802.0	1765.4	4730.0	619	10,434.0
Average egg weight (mg)	1346	39.8	6.4	39.8	20.0	68.1

Regarding reproduction traits, the weight of the total egg mass (here in after referred to as the spawn weight (SW)), the weight (EPW) and number (EPN) of eggs pooled in a 2.5 ml sampling spoon and the spawning week number in the calendar year were all recorded. The spawning week number enabled us to calculate the spawning date (SD) corresponding to the rank of the week number within the spawning period, with discrete values ranging from 1 (for the first week) to 5 (for the 5th and subsequent weeks) within the cohort. The presence of overmature eggs in the spawn was also reported as a potential factor to explain certain reproduction traits. This is an important phenomenon in salmonids that do not spawn naturally under farmed conditions (Escaffre & Billard, 1979). Overmature eggs change in terms of their morphology (McEvoy, 1984) and composition (Craik & Harvey, 1984; Springate et al., 1984), which implies a significant decrease in egg quality.

The weight of coelomic fluid (CF) was determined by subtracting the spawn weight (SW) from the difference between the female weight before (FW) and after (PW) spawning:

CF=(FW‐PW)‐SW.

Average egg weight (EW) was the ratio between the weight of eggs (EPW) and the number of eggs (EPN) contained in the sampling spoon:

EW=EPW/EPN.

The egg numbers in the spawn (EN) were calculated as

EN=SW/EW.

To summarize, the traits analysed were three raw phenotypes, FL, PW and SW, and four derived phenotypes, average egg weight (EW), egg numbers in the spawn (EN), the weight of coelomic fluid (CF) and the spawning date (SD). If any two records were more than four standard deviations from the mean in absolute values, they were considered as outliers and discarded from the study. Thus, the phenotypes of 1346 fish were considered during the study (Table 1).

### 2.3 Genotypes

One thousand three hundred and forty‐six fish (726 and 620 individuals from the C1 and C2 cohorts, respectively) were genotyped for 57,501 SNPs (single nucleotide polymorphism markers) using the Axiom™ Trout Genotyping array (Palti et al., 2015) at the INRAE Gentyane Genotyping Platform. The quality control of genotyped SNPs was performed as described by D'Ambrosio et al. (2019), with particular focus on removing SNPs with probe polymorphism and multiple locations on the genome. Only the 29,799 SNPs with a call rate higher than 0.97, a test of deviation from the Hardy–Weinberg equilibrium with a *p*‐value >0.0001 and a minor allele frequency higher than 0.01 were retained for the analysis. All missing genotypes for the 29,799 SNPs were imputed using family information with FImpute software and default values for all FImpute parameters (Sargolzaei et al., 2014).

### 2.4 Runs of homozygosity

Runs of homozygosity were identified for each fish using the PLINK v1.9 *homozyg* function (Chang et al., 2015) with the options ‘‐‐homozyg‐kb 1000 ‐‐homozyg‐window‐snp 30 ‐‐homozyg‐snp 30 ‐‐homozyg‐gap 1000 ‐‐homozyg‐density 100 ‐‐homozyg‐het 1’. ROH were thus defined by sliding windows with a minimum length of 1 Mb containing at least 30 homozygous SNPs as defined by D'Ambrosio et al. (2019). This minimum number of homozygous SNP was chosen using the formula described by Purfield et al. (2012) in order to limit the number of ROH that might only occur by chance. The maximum gap allowed between two consecutive homozygous SNPs in a run was kept at the default value of 1 Mb. A minimum density of one SNP every 100 kb was considered not to overestimate ROH length and up to one possible heterozygous genotype was permitted for each ROH. All values for parameters used to define ROH were tuned by D'Ambrosio et al. (2019) according to the marker density and the level of recombination along the genome to well estimate the number and size of ROH segments for French rainbow trout populations.

### 2.5 Estimation of inbreeding coefficients

The total inbreeding coefficient (*F_i_*) was calculated as the sum of ROH lengths in an individual *i* (∑Length(ROH*_i_*)) divided by the total length of the autosomal genome covered by SNPs (LGenome):

$$F_{i}=\frac{\sum \text{Length}({\text{ROH}}_{i})}{\text{LGenome}}.$$

The total size of the autosomal genome covered by SNPs (= 1.788 Gb) was calculated as the length of the autosomal genome, removing gaps of more than 1 Mb without any SNP from the total size.

We also derived local inbreeding coefficients, at either the chromosome Omy*k* level or a smaller region *r* scale (20 Mb segment) in order to accurately localize inbreeding events in the genome:

$$F_{\text{Omy}k\_r,i}=\frac{\sum_{k\_r}\text{Length}({\text{ROH}}_{i})}{{\text{LRegion}}_{k\_r}},$$

where ∑*_k_*___ *_r_* Length(ROH*_i_*) is the sum of ROH lengths of an individual *i* on chromosome Omy*k* (or the 20 Mb region *k*_*r* on Omyk), and LRegion*_k_*___ *_r_* is the total length covered by SNPs on the Omyk chromosome or on the *r* region on Omyk.

Furthermore, we derived three other inbreeding coefficients (*F*_G_ *_t_*) depending on the expected generation G*t* (ancient, middle or recent) in which inbreeding started to accumulate.

$$F_{Gt}=\frac{\sum \text{Length}({\text{ROH}}_{i}>n)}{\text{LGenome}},$$

where *t* is the number of generations and *n* the minimal size of a ROH.

The correspondence between the number *t* of past generations and ROH length was derived using the average recombination rate *c* (in Morgan) between markers distant from the threshold value of *n* Mb (with 1 cM corresponding to 600 kb) and the formula *t* = 1/2*c* (D'Ambrosio et al., 2019). Thus, recent inbreeding (*F*_G3_) accumulated on average during the last three generations was calculated as the sum of ROH lengths longer than 10 Mb divided by the total length of the genome covered by SNPs. This ‘3 generation’/‘10 Mb’ ROH length time point was chosen because it is well known that recent inbreeding events occurring during the last three generations play an important role in inbreeding depression effects (Makanjuola et al., 2020). We were also interested by the ‘9 generations’ time point because it corresponded to the start of the breeding programme. On average, this earlier time point corresponds to an ROH size of 3 Mb. In addition, we chose an intermediate time point at 6 generations to better describe the dynamics of inbreeding events throughout the course of the breeding programme. The ROH length corresponding to this intermediate inbreeding (*F*_G6_) was 5 Mb. It should be noted that any one of these temporal inbreeding coefficients could account for all the inbreeding events accumulated since the threshold generation considered, and therefore always contains the most recent inbreeding events. In addition, because the recombination rate varies along the genome, the average dating of size‐specific ROH segments in terms of the number of generations overestimates the age of the most recent ancestor in genomic regions with lower than average recombination rates, but underestimates this age in genomic regions with higher recombination rates. Dating is therefore a very rough proxy used to describe global dynamics at the genome scale without any consideration of absolute dating values at region‐specific scales.

### 2.6 Mixed linear BLUP in animal models

In order to estimate the effects of inbreeding on female size and reproduction traits, two different genetic models were considered: (1) GBLUP— a genomic animal BLUP model with only additive genetic effects, and (2) D_GBLUP— a GBLUP animal model with both additive and dominance genetic effects.

For each of the seven traits studied, the following statistical models were considered to describe the vector of performance **y** of the 1346 phenotyped fish. Genomic BLUP (GBLUP) considering a genomic relatedness matrix **G** (VanRaden, 2007, 2008):

(1) $$y=X\beta +\mathrm{Za}+\sum_{r}b_{r}F_{r}+e$$

where **β**, **a** and **e** are the vectors of, fixed environmental effects, random genetic additive effects and random residual effects, respectively, explaining the performance of all phenotyped animals. **X** and **Z** are the incidence matrices for **β** and **a**, respectively.

For all traits, the cohort fixed effect was considered (two levels). In addition, the spawning week number was introduced as a covariate nested within the cohort for the SW, EN, EW and PW traits. For SW and EN, the presence of overmature eggs was an additional significant fixed effect considered.

The vector **a** includes the breeding values of 1346 phenotyped and genotyped individuals related through the genomic relationship matrix **G**.

The regression coefficients *b_r_* of **y** on the vectors **F_r_** account for inbreeding effects in different ways depending on the model considered. First of all, a single regression on the total inbreeding vector **F** was considered (*r* = 1 in that case). A multiple regression model was then studied and accounted for *r* = 30 vectors of chromosomal inbreeding coefficients *F*_omy_. To finish, a series of *nk* multiple regression analyses was considered to assess the local inbreeding effects for a specific chromosome *k*; in that case, *r* equals the total number *nk* of 20 Mb‐window regions of chromosome *k* in addition to the 29 remaining chromosomes. Depending on the chromosome size, *nk* ranged from 2 (e.g. Omy28) to 4 (e.g. Omy1).

The model accounting for dominance effects (D_GBLUP) was previously described by Vitezica et al. (2016):

(2) $$y=X\beta +\mathrm{Za}+Wd+\sum_{r}b_{r}F_{r}+e$$

where *d* corresponds to the dominance breeding values of 1346 phenotyped and genotyped individuals related through the dominance relationship matrix **D**. **W** is the incidence matrix for *d*. The additive **G** and dominance **D** genomic relationship matrices were built using the *parallelef90* program (Vitezica et al., 2016).

All traits were analysed separately to estimate inbreeding regression coefficients, breeding values and the genetic additive and dominance variances based on the BLUPf90 package (Misztal et al., 2002) using the AIREMLF90 program (Thompson et al., 2005).

## 3 RESULTS

### 3.1 Evaluation of total inbreeding, recent inbreeding and local inbreeding along the rainbow trout genome

Inbreeding coefficients were based on the number and size of ROHs derived for each individual in the population. The average number of ROHs per individual was 61, with an average ROH size of 5.07 Mb. The smallest number of ROHs observed in an individual was 24 and the largest 113. The proportion of large ROH segments (ROH > 10 Mb) corresponding to recent inbreeding events in the population was 11.5%. Inbreeding coefficient values for the complete genome are shown in Table 2 for the full population and its two cohorts according to the number of ancestral generations included.

**TABLE 2:** Summary statistics of total (*F*), old (*F*_G9_), middle (*F*_G6_) and recent (*F*_G3_) inbreeding coefficients in the full population and its two cohorts, C1 and C2

Population	Inbreeding	Mean (%)	SD (%)	Min (%)	Max (%)
Full population (C1 + C2)	*F*	17.27	3.20	7.92	30.05
	*F*_G9_	14.10	3.24	2.56	27.00
	*F*_G6_	11.16	3.24	0.96	24.00
	*F*_G3_	6.56	2.96	0.00	18.51
Cohort C1	*F*	16.76	2.99	8.59	26.55
	*F*_G9_	13.61	3.01	6.06	23.81
	*F*_G6_	10.73	3.05	1.88	21.77
	*F*_G3_	6.13	2.72	0.00	15.14
Cohort C2	*F*	17.87	3.34	7.92	30.05
	*F*_G9_	14.67	3.41	2.56	27.00
	*F*_G6_	11.65	3.39	0.96	24.00
	*F*_G3_	7.07	3.14	0.00	18.51

The average inbreeding level in the population was about 17%, with individual values ranging from 8% to 30%. We observed that 38% of the genome‐wide inbreeding came from the last three generations. In addition, nearly 82% of all inbreeding observed in the C1 and C2 cohorts had been produced since the start of the breeding programme (nine generations earlier). On average, individuals in cohort C2 were more inbred than those in cohort C1 (+6.6% average inbreeding coefficient), this being linked to a few individuals with extreme inbreeding coefficient values (*F* > 25%).

**FIGURE 1:**
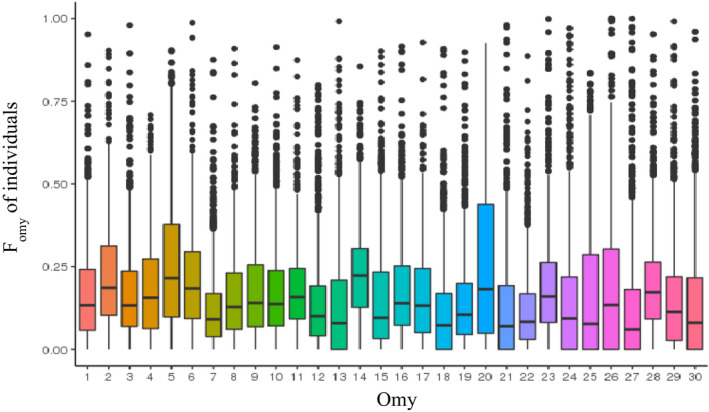
Boxplot of inbreeding coefficients for each of the 30 chromosomes in the 1346 female rainbow trout genotyped

As for inbreeding across chromosomes (Figure 1), we noted that chromosomal inbreeding levels (*F*_omy_) varied considerably along the genome. While the average *F*_omy_ was 17.0%, values ranged from 11.6% for *F*_18_ on Omy18 to 25.1% for *F*_20_ on Omy20. Some individuals had very high *F*_omy_ for a particular chromosome, with values reaching almost 100%. However, because the total *F* of any individual did not exceed 30% (Table 2), an individual with a very high *F*_omy_ on a specific chromosome would have a low *F*_omy_ on the other chromosomes. Details on *F*_omy_ distributions are given in Table S1. With the sole exception of the moderate correlation estimated at 0.61 between *F*_25_ and *F*_30_, the chromosomal inbreeding coefficients (*F*_omy_) were almost uncorrelated (Figure S1).

### 3.2 Heritability and dominance ratios for female size and reproduction traits

With the sole exception of coelomic fluid weight with low heritability value, all traits had intermediate heritability values with estimates higher than 30% for female size traits and between 20% and 30% for reproduction traits, for all genetic models (Table 3). Adding a dominance effect into the genomic model (D_GBLUP) resulted in slight reductions in estimates of genetic variance and heritability when compared to GBLUP. However, the correlations between the additive estimated breeding values from G_BLUP and D_GBLUP models were higher than 0.99 for all traits.

**TABLE 3:** Phenotypic variance (*V*_p_), heritability (*h* ^2^) and dominance ratio (*d* ^2^) of female size and reproduction traits estimated under GBLUP and D_GBLUP models accounting for either the genome‐wide *F* coefficient or the 30 chromosomal inbreeding coefficients *F*_omy_ as covariates (standard errors in brackets). The minimum Akaike information criterion (AIC) value indicates the preferred model for each trait

Traits	GBLUP			D_GBLUP with *F* as covariate				D_GBLUP with 30 *F*_omy_ as covariates			
	*V*_p_	*h* ^2^	AIC	*V*_p_	*h* ^2^	*d* ^2^	AIC	Var	*h* ^2^	*d* ^2^	AIC
PW	107,000 (5033)	0.34 (0.04)	19,196.4	106,740 (5009)	0.33 (0.04)	0.05 (0.04)	19,196.3	105,440 (4977)	0.31 (0.04)	0.07 (0.05)	18,871.9
FL	734.36 (34.9)	0.36 (0.04)	12,502.6	733.9 (34.8)	0.35 (0.04)	0.03 (0.04)	12,503.9	722.9 (34.4)	0.33 (0.045)	0.05 (0.05)	12,324.1
SD	2.074 (0.1)	0.26 (0.04)	11,528.8	2.09 (0.1)	0.26 (0.03)	0.05 (0.01)	11,502.1	2.1 (0.1)	0.26 (0.03)	0.07 (0.01)	10,701.8
CF	1909.9 (80.2)	0.08 (0.03)	12,296.5	1910.0 (77.1)	0.08 (0.03)	0.00 (0.00)	12,270.2	1919.9 (82.9)	0.09 (0.07)	0.04 (0.06)	12,100.6
SW	2746.6 (123.8)	0.28 (0.04)	14,321.3	2756.0 (125.1)	0.27 (0.04)	0.13 (0.05)	14,313.3	2766.0 (128.8)	0.26 (0.04)	0.19 (0.06)	14,092.6
EN	1,760,000 (79,195)	0.27 (0.04)	22,986.1	1,761,000 (79,891)	0.26 (0.04)	0.08 (0.05)	22,984.0	1,775,400 (82,247)	0.25 (0.04)	0.14 (0.06)	22,588.3
EW	37.54 (1.6)	0.26 (0.04)	8606.0	37.6 (1.6)	0.22 (0.04)	0.08 (0.05)	8600.1	38.1 (1.7)	0.23 (0.04)	0.11 (0.06)	8523.4

Abbreviations: CF, coelomic fluid weight; EN, egg number; EW, average egg weight; FL, fork length; PW, postspawning body weight; SD, spawning date; SW, spawn weight.

Considering the genome‐wide inbreeding coefficient as the only covariate in the D_GBLUP model, dominance variance explained about 0%–13% of the phenotypic variance of each trait, the greatest effect being estimated for spawning weight. However, the dominance ratio increased (+25%–107% depending on the trait) when all 30 chromosomal inbreeding coefficients *F*_omy_ were considered as covariates in the D_GBLUP model. In the same model, dominance variance explained about 4% to almost 19% of the phenotypic variance of each trait, the greatest effect still being estimated for spawning weight. The lowest *d* ^2^ estimates concerned female size traits and coelomic fluid weight; they did not differ significantly from 0. Although the *d* ^2^ estimates were moderate for spawning date (5%–7% depending on the D_GBLUP model), they differed significantly from 0. Intermediate dominance ratios (ranging from 8% to 14% depending on the D_GBLUP model) were estimated for egg number and average weight.

Because of their better goodness‐of‐fit (minimum AIC values), the D_GBLUP models were considered to be the reference models to study the impact of inbreeding on performance for all traits, as dominance effects explained at least 5% of phenotypic variance in six out of the seven traits analysed.

### 3.3 Estimating the total inbreeding effect on female size and reproduction performance

Regression coefficients *b* of performance on the total *F* estimated under the D_GBLUP model are presented in Table 4. Significant effects of total inbreeding were only observed for SD and EW. Increased inbreeding raised SD values, that is delayed spawning. A significant reduction of 3.8% and an increase of 12.3% for EW and SD performance were observed with a 0.1 unit increase in the *F* coefficient. Very minor effects (not differing significantly from 0) of total inbreeding were estimated on all other traits, but slight negative trends were observed in all cases.

**TABLE 4:** Regression coefficients *b* for trait performance on the total inbreeding coefficient *F* in the D_GBLUP model

Trait	*b* on *F* (SE)	B* (%)
PW	−175.2 (317.1)	−0.9
FL	−32.0 (25.3)	−0.6
**SD**	**3.15 (1.4)**	**12.3**
CF	−1.52 (44.8)	−0.4
SW	−67.1 (54.7)	−3.5
EN	−491.4 (1336)	−1.0
**EW**	**−15.1 (6.2)**	**−3.8**

Note

B* corresponds to the effect for 0.1 unit increase in the inbreeding coefficient and is expressed as a proportion of mean performance. Traits where inbreeding had significant effects are in bold.

Abbreviations: CF, coelomic fluid weight; EN, egg number; EW, average egg weight; FL, fork length; PW, postspawning body weight; SD, spawning date; SW, spawn weight.

Please note that we use the terms ‘positive’ or ‘negative’ to simply qualify increased or decreased trait values because of an increase in *F*. When we wish to interpret these trends in terms of fitness, the terms ‘favourable’ or ‘unfavourable’ are used.

### 3.4 Local variations in inbreeding effects regarding female size and reproduction traits

**FIGURE 2:**
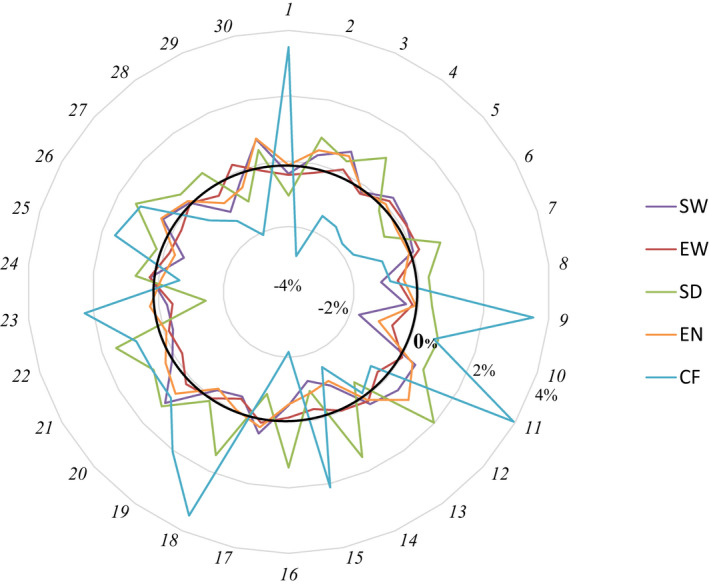
Variations across chromosomes in chromosomal inbreeding effects on reproduction traits. Each point outside the circle represents the 30 chromosomes. The axis of graduation corresponds to B*, that is the effect on trait performance of a variation of +0.1 unit in the chromosomal inbreeding coefficient (expressed as a proportion of mean performance)

When considering the chromosome scale (Figure 2), we observed highly variable effects of local inbreeding on all reproduction traits, even those where total inbreeding had no significant effects. Details of the chromosomal inbreeding effects are presented for all traits in Table S2. For a given chromosome, an increase of 0.1 unit in the inbreeding coefficient corresponded to variations in performance ranging from −2.9% to +3.9% of the trait mean. For some chromosomes, we observed similar negative (e.g. on Omy1, except on coelomic fluid weight) or positive (e.g. Omy20) trends of inbreeding effects on female reproduction traits, while for most of the chromosomes, opposite inbreeding effects were observed on the different traits. Chromosomal inbreeding effects were more variable on coelomic fluid weight (−2.9 to +3.9% of the trait mean, depending on the chromosomes) than on other traits. In particular, the chromosomal inbreeding effects on egg number had a very small amplitude (ranging from −1.1% to 0.9% of the trait mean) and none of them were significant (Figure 2; Table S2). Moreover, we saw a significant and high total inbreeding effect on average egg weight, but with very small chromosomal inbreeding effects ranging from −0.7% to 0.3% of the trait mean. Whatever scale considered (genome‐wide, chromosomal or local scale), very small inbreeding effects were seen to affect female fork length (ranging from −0.2% to 0.3%). Despite these very weak inbreeding effects on this trait, some significant effects were observed on certain chromosomes (see Omy17 and Omy28 in Figure 2) as well as more region‐specific inbreeding effects (see Omy28_r1 in Figure S2).

A few significant chromosomal inbreeding effects (one to four chromosomes, depending on the traits) were estimated for SW, EW, PW and FL. Although we estimated some significant and positive effects of chromosomal inbreeding on the size traits PW and FL, a majority of negative effects were observed for the reproductive traits SW and EW. None of the chromosomal inbreeding effects estimated for SD, EN and CF were statistically different from zero.

Moreover, we did not observe any strong association between chromosomal inbreeding coefficients (*F*_omy_) and the corresponding regression coefficients on performance (Table 5); except for CF, the correlations were not statistically different from zero. Thus, we did not even observe a clear trend towards stronger inbreeding depression with higher *F*_omy_ values. Nevertheless, recent chromosomal inbreeding levels (*F*_G3,omy_) were more markedly negatively associated with the corresponding regression coefficients on performance, particularly for the CF, SW and EN traits.

**TABLE 5:** Pearson correlations between chromosomal inbreeding levels *F*_omy_ and the corresponding regression coefficients *b*_omy_ for each trait, considering either all generations or the three most recent generations

Traits	*r*(*F*_omy_, *b*_omy_)	*r*(*F*_G3,omy_, b_omy,G3_)
PW	−0.098	−0.056
FL	−0.076	−0.097
SD	−0.045	−0.097
CF	**−0.217**	**−0.179**
SW	0.113	−0.273
EN	−0.029	−0.313
EW	0.075	−0.037

Note

Correlations in bold differed significantly from 0.

Abbreviations: CF, coelomic fluid weight; EN, egg number; EW, average egg weight; FL, fork length; PW, postspawning body weight; SD, spawning date; SW, spawn weight.

**FIGURE 3:**
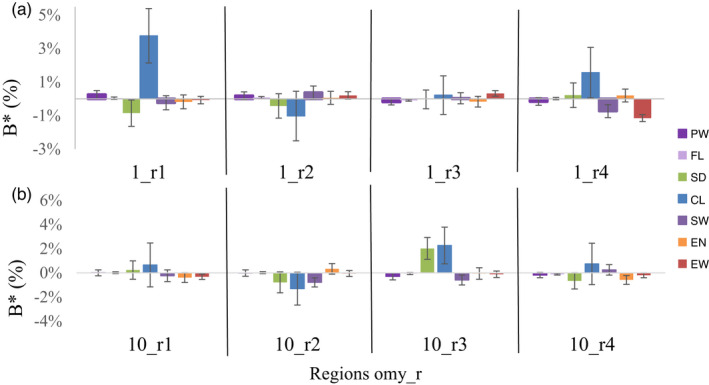
Variations along chromosomes 1 (a) and 10 (b) in local inbreeding effects on female size and reproduction traits. B* corresponds to the effect of a variation of +0.1 unit in the local inbreeding coefficient (expressed as a proportion of trait means)

Zooming at the intra‐chromosomal scale, we also observed highly variable effects of local inbreeding on trait performance whatever the chromosome (F_omy__ *_r_* values are presented in Table S3). We focused our analysis on two chromosomes (Figure 3): Omy1 containing several QTLs on reproduction traits (D'Ambrosio et al., 2020) and Omy10 containing an ROH segment (in the 10_r3 region) shared by diverse rainbow trout populations (D'Ambrosio et al., 2019). Along Omy1 (Figure 3a), highly variable local inbreeding effects were observed, in particular for coelomic fluid weight, with positive effects of the first and last 20‐Mb regions of the chromosome. A unit increase of 0.1 in the local inbreeding coefficient corresponded to performance variations ranging from −1.2% to +3.8% of the trait means. Except for average egg weight, which had a significant positive inbreeding effect on region 1_r3, local inbreeding coefficients exerted no significant effects on the two intermediate regions of Omy1. In addition to the positive inbreeding effect of the 1_r4 region on CF, significant negative effects of inbreeding in this 1_r4 region were observed for EW and SW. We also observed highly variable effects of local inbreeding along the Omy10 chromosome (Figure 3b). The effects were small and not statistically different from 0 in the first and last regions of Omy10. However, local inbreeding in the second and third regions of Omy10 exerted significant effects, but only on spawn weight (decreased performance) and spawning date (increased performance).

### 3.5 Variations over generations in inbreeding effects on female size and reproduction traits

**FIGURE 4:**
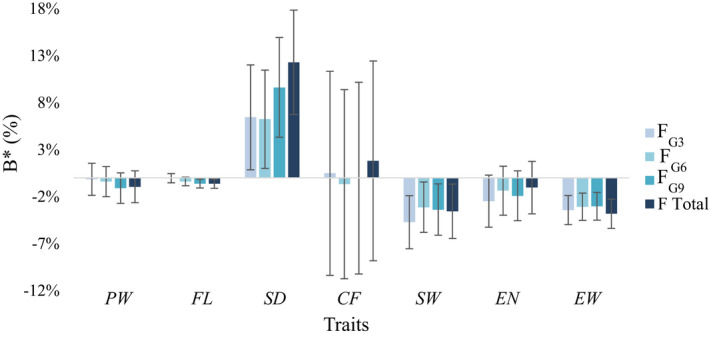
Variations over cumulated generations of ancestral inbreeding effects on female size and reproduction traits. B* corresponds to the effect of a variation of +0.1 unit in the inbreeding coefficient (expressed as a proportion of trait means)

Genome‐wide inbreeding effects over generations were generally very small, except for SD, SW and EW (Figure 4). We analysed inbreeding effects due to total inbreeding (*F*) accumulated in the population, but also due to inbreeding events that had accumulated over the past nine generations that we qualified as old inbreeding (*F*_G9_), or over just the past three generations that we qualified as recent inbreeding (*F*_G3_). Comparing this recent time point to the intermediate point of six generations (*F*_G6_), we are able to assess inbreeding events that had occurred between generations 4 and 6. Regarding average egg weight, the effects of recent inbreeding, as well as those accumulated since older generations, were significantly negative. Similar trends were observed for spawn weight although the inbreeding effects did not differ significantly from 0. All temporal inbreeding effects showed clear positive trend on spawning date, but they did not differ significantly from 0, except for the total inbreeding effect. Inbreeding effects mainly appeared to be due to recent inbreeding (*F*_G3_) for EW and SW, whereas they appeared to be due to both recent and older inbreeding events for SD.

**FIGURE 5:**
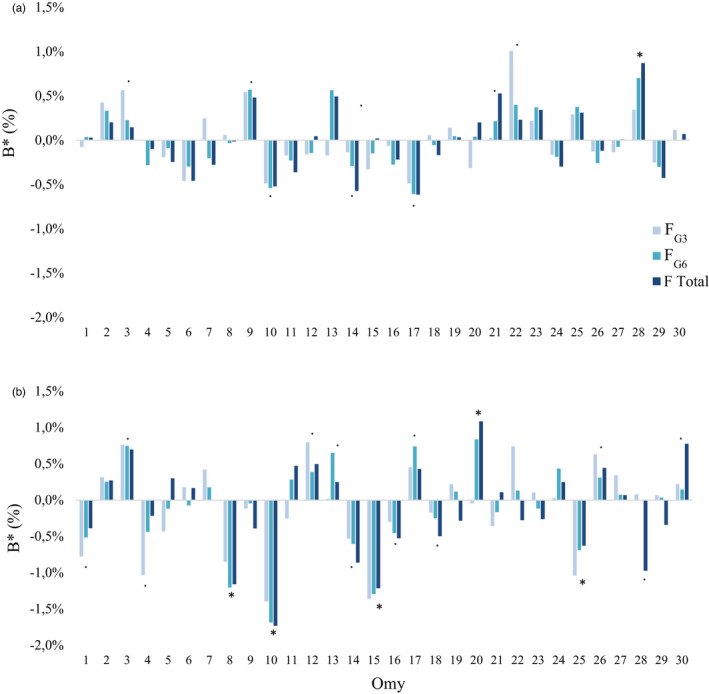
Variations in inbreeding effects on traits postspawning weight (a) and spawn weight (b) along chromosomes and over generations. * indicates a significant inbreeding effect; . indicates a inbreeding effect trend

As for both temporal and chromosomal inbreeding effects along the genome, we observed highly variable effects for all traits (Table S2). We focused our analysis on postspawning body weight and spawn weight (Figure 5) in order to highlight the point that effects could be revealed at temporal and/or chromosomal scales even when total inbreeding effects were null at the genome scale. Indeed, a variation of 0.1 unit in the inbreeding coefficient corresponded to performance variations ranging from −3.9% to +4.6%, depending on the trait, the chromosome and the number of generations used to evaluate the inbreeding effect.

An impact of recent inbreeding (*F*_G3_) was considered to be important when, in absolute values, the recent inbreeding effect was equal to or higher than the other temporal inbreeding effects. Important impacts of older inbreeding events were considered when nonzero values of total (*F*) and old (*F*_G9_) inbreeding effects were estimated but the recent inbreeding effect was estimated to be null. Applying these definitions, chromosomal inbreeding effects appeared to be largely explained by recent inbreeding. Indeed, 50% (all traits gathered together, otherwise 40%–67% depending on the trait) of chromosomes with nonzero B* estimates (above the threshold of 0.5% of the trait mean) indicated an important impact of recent inbreeding, while 33% of chromosomes (21%–50% depending on the trait) displayed important effects of older inbreeding events.

Six scenarios of inbreeding evolution over generations were observed depending on the trait and chromosome. We present them below, from the most common to the least frequent.

The first scenario corresponded to negative effects of recent inbreeding (as shown in Figure 5 for PW on Omy6; SW on Omy4). The second scenario consisted in positive effects of recent inbreeding (e.g. Figure 5 for PW on Omy22; SW on Omy12). The third scenario was associated with negative effects of older inbreeding events (as shown in Figure 5 for SW on Omy28). The fourth scenario was related to positive inbreeding effects in old generations (e.g. Figure 5 for PW on Omy13; SW on Omy30), while the fifth scenario corresponded to observations of negative inbreeding effects accumulated from old to recent generations (as shown in Figure 5 for PW on Omy11; SW on Omy16). The last scenario was related to positive inbreeding effects accumulated from old to recent generations (as shown in Figure 5 for PW on Omy28).

## 4 DISCUSSION

Because inbreeding effects are mainly due to dominance genetic effects, we first of all quantify and discuss the importance of dominance effects on performance.

### 4.1 Genome‐wide dominance effect on female size and reproduction traits

Dominance variance arises from heterozygotes deviating from the mean of the two homozygotes. Loci with overdominant alleles (i.e. inducing a heterozygote advantage) cause high dominance genetic variance in populations (Haldane, 1947), whereas the partial dominance hypothesis predicts that most genetic variations will be additive when the mutations are incompletely recessive (Houle et al., 1992). Therefore, the partitioning of genetic variance into components of additive variance and dominance variance may help to assess the relative contributions of genetic load and heterozygote advantage to genetic variations in performance.

At least 4% of the phenotypic variances of the female size and reproduction traits were explained by dominance effects in our study. The high dominance ratios (up to 19%) we estimated for reproduction traits (particularly spawn weight and egg number) when compared to size traits may have corresponded to a larger number of loci with overdominant alleles acting on female fecundity.

As far as we know, the literature is very scarce regarding estimates of variance dominance in fish, except for growth and size traits. Based on pedigree estimates and large number of families composed of about 100 full‐sibs in three populations of rainbow trout, the *d* ^2^ for rainbow trout body weight (Pante et al., 2002) ranged from 0% to 21% depending on the populations. With a similar family structure in four populations of Atlantic salmon, Rye and Mao (1998) indicated that dominance and additive‐by‐additive epistasis variances were equal to or greater than the additive variance for body weight, with ratios ranging from 2% to 9% and 13% to 16% of phenotypic variance, respectively.

In a recent study based on genomic information on Nile tilapia, Joshi et al. (2020) showed that nonadditive genetic effects were negligible regarding body length and were explained by additive‐by‐additive epistasis rather than dominance for body weight at harvest. They reported that under pedigree analysis, the additive‐by‐additive epistasis component was confounded with the dominance component. Our low estimates of dominance ratios for postspawning body weight and female fork length therefore appear to be in relatively good agreement with these previous findings in different fish species.

Regarding *d* ^2^ estimates for reproduction traits, we could only compare our estimates to previous studies in terrestrial livestock species where dominance variance ratios were estimated at around 2% for pig litter size (Vitezica et al., 2016, 2018) and 0%–10% for layers on reproduction traits (i.e. egg production, egg colour, egg weight and yolk weight) (Heidaritabar et al., 2016; Misztal & Besbes, 2000).

To the best of our knowledge, our study is the first to have looked at the partitioning of phenotypic variance when performance is corrected for chromosomal inbreeding effects rather than a genome‐wide inbreeding effect. The estimates of dominance variance were markedly higher when applying a D‐GBLUP model with multiple corrections for all the 30 chromosomal inbreeding coefficients (*F*_omy_) compared to simple regression on the genome‐wide inbreeding coefficient (Table 3). While this important increase in dominance variance captured by the model using the combined effects of all *F*_omy_ was mainly associated with a reduction in residual variances for reproduction traits, it was generally associated with a reduction in additive genetic variance among female size traits. The reduction in residual variances corresponded to a better fit of the model to the data (minimum AIC value) but did not provide any clues regarding the mechanisms that underlie dominance for reproduction traits. The reduction in genetic variance for size traits could perhaps be explained by additive‐by‐additive epistasis effects captured in the dominance effect when regressing performance on all *F*_omy_, which could confirm the pseudo‐overdominance hypothesis affecting some loci (Ohta & Kimura, 1969).

### 4.2 Genome‐wide inbreeding effect on female size and reproduction traits

The mean levels of recent (6.6%) and total (17.3%) inbreeding in the ninth generation of selection of the population under study were slightly higher than previous estimates (5.8% and 16.6%, respectively) in the eight generation (D'Ambrosio et al., 2019). This corresponds to an increase in inbreeding rate ∆*F* of 0.7% in one generation, which is below the 1% limit targeted under the optimal contribution selection procedure recommended for fish breeding programmes (Skaarud et al., 2014).

Nevertheless, inbreeding levels were quite high when compared to those that can be observed in terrestrial livestock (D'Ambrosio et al., 2019). The main objective of our study was therefore to quantify inbreeding effects at different genomic scales (all genome, chromosome or region‐wide), as well as at different time periods. Considering that the positive effects of inbreeding we observed on spawning date were in fact unfavourable because they delayed the moment of spawning, we can sum up our results by saying that unfavourable or null effects of genome‐wide inbreeding were estimated on all female size and reproduction traits.

These results corroborate widespread observations of inbreeding depression on fitness‐related traits. First of all, the significant negative inbreeding effect observed on average egg weight was undoubtedly unfavourable as large egg size is advantageous in terms of fitness (Einum & Fleming, 1999; Hutchings, 1991); egg size has a direct effect on the body size of offspring at emergence and hence on fry survival. As for spawning date, delayed spawning in a wild context is considered to be favourable to fitness when predators and competition exist between either females for spawning space (Essington et al., 1998) or offspring for territory (Brännäs, 1995). Without predators, however, early spawning may be advantageous because offspring can grow faster than progeny born later because of their access to the best available feeding habitat (Brännäs, 1995; Einum & Fleming, 2000). We can hypothesize that spawning date is always under stabilizing selection when environmental conditions remain unchanged, as it has been suggested by Ford et al. (2006) evaluating long‐term changes in a naturally spawning coho salmon population after several decades of intensive hatchery supplementation. Their study showed an optimum run timing observed with fish that returned to their native creek at either end of the run timing distribution producing fewer offspring compared with fish that returned in the middle of the distribution. Under farming and selection conditions, early spawning is expected to be favourable to fitness because offspring are then larger and have a greater chance of being selected (Chevassus et al., 2004). In addition, delayed spawning leads to larval rearing at warmer temperatures, which is unfavourable for some species of salmonids and may increase fry mortality (Crozier & Zabel, 2006; Smith et al., 2003).

It therefore makes sense to interpret the significant positive inbreeding effect observed on spawning date in our commercial selected line as being unfavourable in terms of fitness.

It should be noted that we did not observe any significant genome‐wide effects of inbreeding for size traits, although such effects have generally been observed in studies on salmonids and particularly on rainbow trout. In terms of postspawning weight in rainbow trout, Su et al. (1996) estimated an inbreeding depression of 3.9% per 0.1 unit increase in inbreeding. Pante et al. (2001) estimated a reduction in body weight at harvest that ranged from −1.7% to −5.0% per 0.1 unit increase in inbreeding, depending on the population, the highest values being very similar to earlier estimates for adult body weight (Gjerde et al., 1983; Kincaid, 1983). All these estimations were higher than ours with respect to female postspawning weight. Our estimate (−0.9% per 0.1 unit increase in *F*) was closer to an estimate of 2‐year weight in four Atlantic salmon populations, ranging from −0.6% to −2.6% per 0.1 unit increase in *F* (Rye & Mao, 1998). Based on genomic information, Waters et al. (2020) found that inbreeding did not affect female weight and fork length in two hatchery lines of Chinook salmon derived from the same source. Inbreeding and its potential effects on growth depend on a variety of factors, including environmental conditions (Armbruster & Reed, 2005), that may explain the variable effects observed across salmonid populations.

Concerning inbreeding effects on reproduction traits, Waters et al. (2020) showed in Chinook salmon that inbreeding did not affect fecundity but delayed spawn timing by 1.75 days per one standard deviation increase in *F*. While we also observed a significant delay in spawn timing (with an increase of +12% in SD per +0.1 unit increase in *F* in our trout population), we detected some inbreeding depression effects on spawn weight (−3.5% per +0.1 unit in *F*) and EN (−1.0% per +0.1 unit in *F*), although these effects were not statistically significant. The inbreeding effect on egg number was modest when compared to the estimate of −6.1% for EN per +0.1 unit increase unit in *F* derived from an experimental line of rainbow trout (Su et al., 1996). This negative inbreeding effect is likely to be unfavourable in terms of fitness, because a large number of eggs is expected to produce a large number of juveniles, at least if egg quality is not altered when egg production is high. While a slight negative phenotypic correlation was observed in our study population between average egg weight and egg number, a null genetic correlation had been estimated between them (D'Ambrosio et al., 2020), which corroborates earlier results in the rainbow trout (Su et al., 1996). Assuming that egg weight is a good predictor of egg quality, we can hypothesize that the fall in EN and EW values observed with increased inbreeding levels would be unfavourable to fitness in rainbow trout populations.

All these varying results across studies could be due to species differences, environmental differences (Armbruster & Reed, 2005), different degrees of inbreeding and performance or population management factors. For example, the effect of inbreeding may be undetectable on fitness traits if the rate of inbreeding is slow (Wang et al., 1999).

### 4.3 Local inbreeding effects on female size and reproduction performance

Because of the marked variations in inbreeding levels we observed along the genome, it was indeed intuitive to expect stronger and more variable effects of local inbreeding than those of genome‐wide inbreeding. The first question we tried to address was therefore whether a higher chromosomal inbreeding coefficient corresponded to greater inbreeding effects on performance? The general answer was that no strong associations were observed between the chromosomal inbreeding level and the corresponding regression coefficient impacting performance. However, for all traits, higher recent chromosomal inbreeding levels (*F*_G3,omy_) have been associated with negative effects on performance (Table 5), although these trends were only clear for CF, SW and EN. This observation is consistent with the literature, where it has been said that recent inbreeding has more deleterious effects than older inbreeding (Doekes et al., 2019).

When focusing on chromosomal and region‐specific inbreeding effects on performance, we observed a mixture of negative and positive effects on each trait. This phenomenon had been also shown in a wild bird population by Botero‐Delgadillo et al. (2020) through a correlation study between heterozygosity and fitness traits. Because region‐specific inbreeding effects are not exclusively unfavourable, the global unfavourable or null effects observed for genome‐wide inbreeding should however correspond to an accumulation of larger numbers and/or stronger impacts of unfavourable local inbreeding effects. For example, *F*_10_ had a significant negative effect on spawn and average egg weight (Figure 3), but to the best of our knowledge, no QTL has been found to be related to female reproduction traits on Omy10. As for the favourable effects of inbreeding, we should underline the significant positive effect of *F*_28_ on female length and weight (Figure S2), which may be linked to a putative QTL on Omy28 and which explained about 1.5% of genetic variance in body weight at 18 months in a Chilean rainbow trout line (Neto et al., 2019). Two candidate genes have been identified in this QTL region (located between 20.6 and 21.3 Mb): G protein‐coupled receptor‐54 like 1 (GPR54L1) and early growth response 1 (EGR1), which plays an important role in growth processes (Aljada et al., 2002; McKee et al., 1997). These results may explain the positive effect of inbreeding on the second region r_2_ of Omy28 with respect to female size traits in our French line. Increased size performance (PW and FL) has undoubted favourable effects in terms of fitness among farmed populations. Firstly, large body weight is one of the main selection goals for breeders, and secondly, large body size has been positively correlated with fish survival and reproduction in several studies (Foote, 1990; Huang & Gall, 1990; Pollock et al., 2007; Quinn & Peterson, 1996).

When focusing on local windows of 20 Mb within a chromosome, we also observed strong variations in inbreeding effects for all traits. For example, and as shown in Figure 3, for average egg weight and Omy1 we observed a small but significant favourable effect on region r3 (from 40 to 60 Mb), but a clearly unfavourable inbreeding effect on region r4 (60 to 80 Mb). Regarding the latter case, several QTLs for egg weight have been detected on the same dataset (D'Ambrosio et al., 2020) and deleterious haplotypes have been observed in another French line (Fraslin et al., 2020) that might explain inbreeding depression.

These observations convinced us that the study of local inbreeding is of prime importance to identifying genomic regions and genes with a major impact on inbreeding depression or, on the contrary, regions where inbreeding should be favoured. Further study of inbred regions will help us to highlight selective sweeps and genomic signatures of selection (Aramburu et al., 2020), thus retracing the evolutionary history of the population. Although we have considered the identified QTLs in splitting chromosomes in 20‐Mb regions, we probably cut some ROHs into two segments and the QTLs may not locate in the same region as the majority of the ROHs. Therefore, the search for regions where increased inbreeding is associated with impaired phenotypic performance is not trivial. The use of sliding windows of small size rather than fixed 20‐Mb windows may overcome this issue and help to better estimate local inbreeding effects.

### 4.4 Origin and evolution of inbreeding along the genome

The second objective of our study was to propose underlying mechanisms that might explain our observations regarding temporal and spatial inbreeding effects. All hypotheses relate to underlying mechanisms associated to nonadditive genetic effects, but their long‐term implications are not the same. For the overdominance hypothesis, selection would favour heterozygote states at multiple loci, so that mutations would be maintained by mechanisms related to balancing selection. Under the partial dominance hypothesis, the selection of inbred individuals with good performance to become reproducers would purge any deleterious alleles generated by mutations (Kristensen & Sørensen, 2005).

We have identified six scenarios that describe the evolution of local inbreeding effects over time. Here, we will connect these scenarios with some hypotheses including the three that underlie inbreeding depression, that is partial dominance, overdominance and pseudo‐overdominance.

Scenarios with inbreeding effects due to recent inbreeding are the most common, particularly when the effects are negative, as has also been shown in cattle (Doekes et al., 2019; Makanjuola et al., 2020). Our first scenario, corresponding to the unfavourable effects of recent inbreeding, might be explained by any of the three hypotheses underlying inbreeding depression. Under the partial dominance hypothesis, this means that recent deleterious alleles have not yet been purged. Under the overdominance and pseudo‐overdominance hypotheses, inbreeding depression is not subject to any allelic purge (Charlesworth & Willis, 2009). The second scenario corresponds to a favourable effect of recent inbreeding. This can be explained by one favourable allele or underdominance, which corresponds to the disadvantages of a heterozygous genotype regarding reproductive success (the advantage of both homozygous genotypes) that increased the frequency of homozygotes (Reed et al., 2013).

Scenarios 3 and 4 concern the regions where we observed older inbreeding effects on performance which were either unfavourable (scenario 3) or favourable (scenario 4).

Scenario 4 corresponds to either an underdominance phenomenon or to the purging of deleterious alleles (under the partial dominance hypothesis), leading to the fixation of favourable alleles and improved fitness associated with domestication and/or selection phenomena. Purging tends to regress inbreeding depression towards zero because homozygosity in some genomic regions is no longer unfavourable (Boakes et al., 2007). Scenario 3 relates to a lack of purging and local recombination events under the three hypotheses of inbreeding depression, leading to the maintenance of old inbreeding with unfavourable effects on performance (Hedrick, 1994). This lack of effective purging can be explained by alleles, which have only minor unfavourable effects on performance. Indeed, the purging mechanisms are effective in the case of alleles with severely deleterious or even lethal effects (Charlesworth et al., 1990; Wang et al., 1999), which are purged at a faster rate than mildly deleterious alleles. In the case of rare alleles with minor effects, deleterious alleles can be eliminated over hundreds or even thousands of generations (Hedrick, 1994; Hendry et al., 2011; Lande & Schemske, 1985; Larsen et al., 2011). As a direct consequence of purging, populations with a long history of inbreeding are likely to be less affected by inbreeding depression than others with similar levels of inbreeding because they have had more opportunity to purge deleterious alleles than those with recent inbreeding history (Day et al., 2003; Ehiobu et al., 1989). As we observed quite frequently under scenario 3, we can suppose that the bottleneck associated with initial steps in rainbow trout domestication and selection have increased the mutational load of the population and caused an accumulation of deleterious alleles without it being possible to purge them all (Bosse et al., 2019).

The least frequent scenarios with unfavourable (scenario 5) or favourable (scenario 6) effects from old to recent inbreeding correspond to genomic regions subjected to a combination of events observed in the previous scenarios. Recent inbreeding effects are observed, but a lack of purging of deleterious alleles or a low recombination rate in those specific regions may maintain unfavourable old inbreeding effects.

For all the scenarios revealing unfavourable effects of inbreeding, it is not possible to differentiate the three hypotheses underlying inbreeding depression. Nevertheless, the high estimates of dominance variance for some traits may indicate that these traits might be more affected by overdominant loci.

### 4.5 Variable evolution of inbreeding effects depending on traits

Inbreeding effects vary considerably along the genome but also from one trait to another because of different histories during domestication and selection processes.

Size traits have been strongly selected since species domestication. The purging of deleterious alleles has already occurred and some favourable alleles for growth function have been selected, thus explaining the positive effects of old inbreeding such as those observed on Omy28. Favourable effects of recent inbreeding are also observed on Omy22, although no QTL seems to have been identified yet for body weight or size on this chromosome, apart one linked to head yield in a different French rainbow trout population line (Blay et al., 2021).

Globally, we found that all chromosomal inbreeding effects were low for female length and weight, as were the estimates of dominance variance which did not exceed 7%. We therefore believe that the partial dominance hypothesis is the best to explain inbreeding depression affecting female size traits.

By contrast, the quantity of coelomic fluid (CF) is not a trait under direct selection. We observed the highest chromosomal inbreeding effects for this trait and a general trend towards an accumulation of recent and older chromosomal inbreeding effects. We can therefore assume that inbreeding depression for this trait is due to an accumulation of deleterious mutations. There are no mentions in the literature regarding the quantity of coelomic fluid, but its composition appears to be linked to progeny survival (Inanan, 2020; Kobayashi et al., 2001). Nevertheless, our CF results need to been taken with caution as CF was a trait derived indirectly through the use of three weight measurements.

For spawning date, we observed both a significant genome‐wide inbreeding effect and marked chromosomal inbreeding effects. As a result of the low estimate for the dominance variance ratio (5%), the overdominance hypothesis could not explain a large part of inbreeding effects and the hypothesis of partial dominance should be preferred to explain inbreeding depression.

A significant genome‐wide inbreeding effect was also observed for average egg weight, while chromosomal inbreeding effects were null and the dominance ratio was moderate (8%). We therefore postulate an accumulation of small negative inbreeding effects due to slightly unfavourable alleles, which is consistent with the partial dominance hypothesis underlying inbreeding depression for egg weight.

As for the fecundity traits spawn weight and egg number, we observed quite similar profiles of temporal and local inbreeding effects, consistent with their high genetic correlation (D'Ambrosio et al., 2020). Although we did not estimate any significant genome‐wide inbreeding effect for these two traits, numerous chromosomes displayed significant and high inbreeding effects. In addition, the dominance variance ratios were quite high (19% for SW and 14% for EN). We can therefore assume that the inbreeding depression observed for fecundity traits could be explained by both the partial dominance and overdominance hypotheses.

## 5 CONCLUSION AND IMPLICATIONS

To conclude, we observed very variable inbreeding effects on female size and reproduction traits along the genome and over generations. These results suggest that local inbreeding can strongly impact performance without observing any effects of genome‐wide inbreeding. In general, and as might usually be expected, we observed either null or unfavourable effects of genome‐wide inbreeding on female size and reproduction traits. However, some favourable effects of local inbreeding were also observed and could be explained by either the selection of favourable homozygotes in the population or an underdominance effect of the heterozygotes. Partial dominance appeared to be the main hypothesis explaining inbreeding depression for all the traits studied, although regarding fecundity traits (spawn weight and egg number), the overdominance hypothesis could also play a significant role in inbreeding depression. Future studies need to focus on the evolution of inbreeding effects near regions under intense directional selection. The limitation of using ROH‐based metrics to identify a region associated with inbreeding depression is that a region with long stretches of homozygosity may contain multiple ROH genotypes with variable effects on the phenotype of interest. Further investigations are therefore necessary to determine which specific ROH genotypes result in unfavourable performance.

These new findings offer important keys to the future management of breeding programmes by considering the local genomic scale and not just the genome‐wide scale to allow inbreeding in areas where its impact is favourable, and limit it when its effect is deleterious on traits of interest. To achieve this, two options have been advocated to reduce inbreeding depression; purging induced by deliberate inbreeding and genetic rescue.

## CONFLICT OF INTEREST

The authors declare no conflicts of interest.

## Supporting information

Supplementary Material

## Data Availability

The data that support the findings of this study are available from the breeding company ‘Viviers de Sarrance’ but restrictions apply to the availability of these data, which were used under license for the current study, and so are not publicly available. The data can be made available for reproduction of the results from Florence Phocas (florence.phocas@inrae.fr) and Ana Acin‐Perez (ana@sarrance.com) on request via a material transfer agreement and with permission of the breeding company ‘Viviers de Sarrance’.
