# Supplementary material for: Temporal and region‐specific variations in genome‐wide inbreeding effects on female size and reproduction traits of rainbow trout
Source: Evol Appl. 2021 Oct 21;15(4):645–62. doi: 10.1111/eva.13308 (PMC9046919; doi:10.1111/eva.13308)
Supplement: Supplementary file 1 — Supplementary Material [file EVA-15-645-s001.doc]

**Supplementary Informations**

**Supplementary tables :**

**TABLE S1 Summary statistics of chromosomal inbreeding coefficients for F_omy_ values in the full population of rainbow trout (1346 individuals).**

| **F_omy_** | **Mean (%)** | **Q1 (%)** | **Median (%)** | **Q3 (%)** |
| --- | --- | --- | --- | --- |
| F_1_ | 16.5 | 5.78 | 13.3 | 24.2 |
| F_2_ | 22.2 | 10.3 | 18.6 | 31.2 |
| F_3_ | 16.8 | 6.93 | 13.3 | 23.6 |
| F_4_ | 18.5 | 6.28 | 15.6 | 27.3 |
| F_5_ | 24.7 | 9.78 | 21.5 | 37.8 |
| F_6_ | 20.9 | 9.32 | 18.4 | 29.5 |
| F_7_ | 12.1 | 3.85 | 9.09 | 16.9 |
| F_8_ | 15.8 | 6.07 | 12.8 | 23.1 |
| F_9_ | 17.5 | 6.84 | 14.0 | 25.5 |
| F_10_ | 17.7 | 7.09 | 13.7 | 23.8 |
| F_11_ | 18.4 | 9.19 | 15.8 | 24.5 |
| F_12_ | 14.1 | 4.09 | 10.0 | 19.2 |
| F_13_ | 12.9 | 0 | 7.92 | 20.9 |
| F_14_ | 22.2 | 12.7 | 22.3 | 30.5 |
| F_15_ | 15.8 | 3.25 | 9.53 | 23.4 |
| F_16_ | 17.9 | 7.29 | 13.9 | 25.2 |
| F_17_ | 16.4 | 5.07 | 13.2 | 24.4 |
| F_18_ | 11.6 | 0 | 7.26 | 16.9 |
| F_19_ | 14.3 | 4.49 | 10.5 | 19.9 |
| F_20_ | 25.1 | 4.86 | 18.2 | 43.8 |
| F_21_ | 12.8 | 0 | 7.00 | 19.2 |
| F_22_ | 11.8 | 3.0 | 8.33 | 16.8 |
| F_23_ | 19.2 | 8.14 | 16.0 | 26.2 |
| F_24_ | 15.0 | 0 | 9.35 | 21.9 |
| F_25_ | 18.1 | 0 | 7.70 | 28.6 |
| F_26_ | 19.2 | 0 | 13.4 | 30.3 |
| F_27_ | 12.4 | 0 | 6.03 | 18.1 |
| F_28_ | 19.6 | 9.19 | 17.25 | 26.4 |
| F_29_ | 15.2 | 2.70 | 11.3 | 21.9 |
| F_30_ | 15.6 | 0 | 8.04 | 21.6 |

**TABLE S2** Details of chromosomal inbreeding effects (standard errors in brackets) on the seven traits studied and 30 chromosomes (Omy). We considered chromosomal inbreeding effects over generations with total (F_omy_), middle (F_omy,G6_) and recent (F_omy,G3_) inbreeding coefficients in the full population.

**TABLE S3** Summary statistics of local inbreeding Fomy_region values, for regions of 20Mb on specific omy1, omy10 and omy28, in the 1346 rainbow trout genotyped.

| **FOmy_region** | **Mean (%)** | | **Q1 (%)** | **Median (%)** | **Q3 (%)** |
| --- | --- | --- | --- | --- | --- |
| F1_r1 | 11.9 | 0 | | 0 | 15.0 |
| F1_r2 | 16.5 | 0 | | 7.1 | 27.5 |
| F1_r3 | 20.3 | 0 | | 5.8 | 26.4 |
| F1_r4 | 17.4 | 0 | | 9.8 | 24.5 |
| F10_r1 | 13.4 | 0 | | 9.6 | 16.2 |
| F10_r2 | 21.1 | 0 | | 11.4 | 27.9 |
| F10_r3 | 20.2 | 0 | | 16.8 | 29.0 |
| F10_r4 | 6.9 | 0 | | 0 | 0 |
| F28_r1 | 13.8 | 0 | | 6.1 | 22.4 |
| F28_r2 | 25.2 | 11.1 | | 23.8 | 37.8 |

**Supplementary figures :**

**
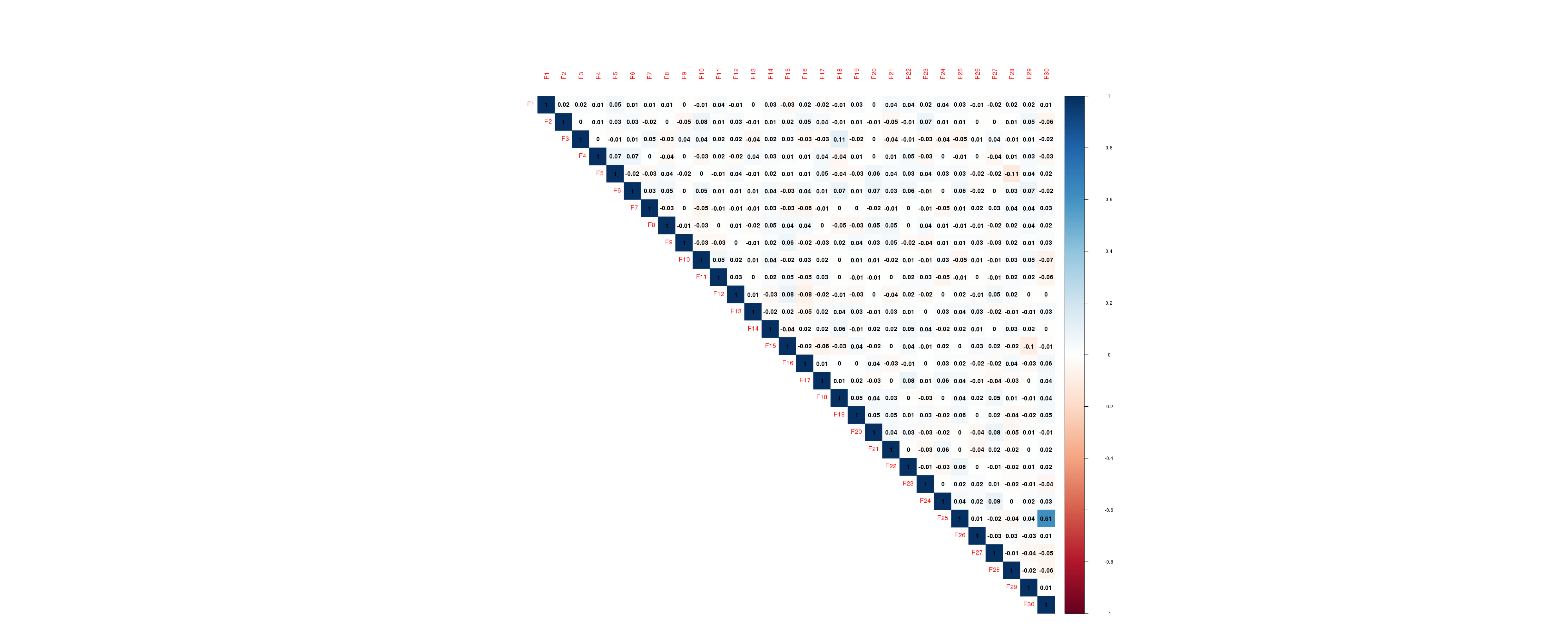
**

**FIGURE S1** Correlations between chromosomal inbreeding coefficients (F_omy_). Except for the correlation between F_25_ and F_30_ (p-value>2.26^e^10-16), all correlations were not significant (p-value >0.05).


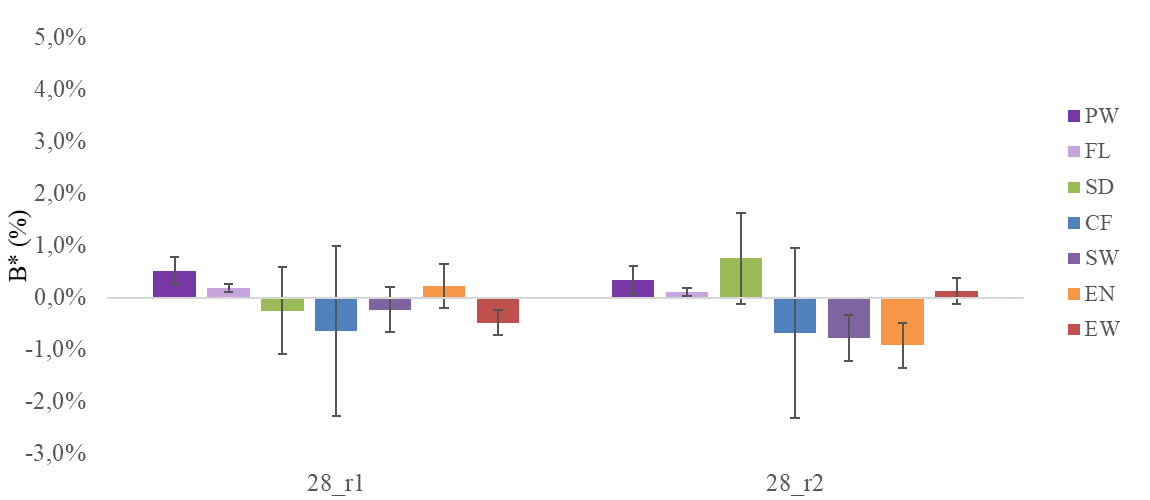


**FIGURE S2**  Variations along chromosome 28 in local inbreeding effects on female size and reproduction traits.
